# Malonic acid suppresses lipopolysaccharide-induced BV2 microglia cell activation by inhibiting the p38 MAPK/NF-κB pathway

**DOI:** 10.1080/19768354.2021.1901781

**Published:** 2021-03-18

**Authors:** Hana Lee, Jun-Ho Jang, Seok-Jun Kim

**Affiliations:** a Department of Integrative Biological Sciences & BK21 FOUR Educational Research Group for Age-associated Disorder Control Technology, Chosun University, Gwangju, Republic of Korea; b Department of Biomedical Science, Chosun University, Gwangju, Republic of Korea

**Keywords:** Malonic acid, neurodegenerative diseases, microglia, inflammation, p38 MAPK/NF-κB pathway

## Abstract

An inflammatory reaction caused by the activation of microglia in the brain can lead to neurodegeneration and cause diseases, such as Alzheimer’s and Parkinson’s disease. The regulation of inflammation can aid in preventing the development of neurodegenerative disease. Malonic acid has a variety of biological activity. The effects of malonic acid on microglia are not currently well known. Therefore, in this study, we investigate the effects of inflammation of malonic acid in BV2 microglia cells. As a result, we demonstrated that malonic acid on LPS-treated microglia decreased pro-inflammatory responses and mechanisms of the p38 MAPK/NF-κB pathway. Inflammatory mediators significantly decreased the LPS-induced production of nitric oxide and reactive oxygen species. Pro-inflammatory cytokines of IL-6 suppressed gene expression. In addition, the protein expression of NF-κB decreased at the nucleus, as did the protein expression of activated phosphorylated IκB-α, which is an NF-κB regulator-related protein. The expression of phosphorylated p38, a mediator of inflammatory cytokines, was regulated. Therefore, our results indicate that malonic acid has anti-inflammatory effects and may be a potential therapeutic candidate for neuroinflammatory diseases.

## Introduction

Microglia, a type of resident macrophage located in the brain, are involved in the innate immune response of the central nervous system (CNS) (Ransohoff and Brown 2012). Under normal conditions, microglia play a role in homeostasis regulation and host defense (Hickman et al. 2018). However, when microglia are activated by injury and infection, they produce various pro-inflammatory mediators (free radicals), including nitric oxide (NO), reactive oxygen species (ROS), and cytokines, such as interleukin (IL)-1β, IL-6, and tumor necrosis factor (TNF)-α (Kreutzberg 1996; Hanisch 2002). Activated microglia cause neuroinflammation related to neurodegenerative disorders, thus, there is a possibility that they play a role in the development of Alzheimer’s disease (AD) and Parkinson’s disease (PD), among others (Olson and Miller 2004; Lucas et al. 2006; Hansen et al. 2018; Ho 2019). Therefore, the reduction of activated microglia may prove to be an effective therapeutic method for the treatment of neurodegenerative diseases.

Recently, a number of studies have focused on the anti-inflammatory agents of natural compounds (Azab et al. 2016). Malonic acid, also known as Propanedioic acid, is a dicarboxylic acid with the structure CH_2_(COOH)_2_ that is found in plants and vegetables (Bentley 1952; Priecina and Karklina 2015). Various studies have demonstrated that malonic acid exerts beneficial effects on fruit growth and chemical synthesis. Recent studies have also shown that bis-malonic acid fullerene derivative can inhibit IL-6 expression in bone marrow-derived mast cells (Funakoshi-Tago et al. 2016). However, the mechanism underlying the anti-inflammatory effects of malonic acid in microglia are not yet fully understood. Therefore, we focused on the role of malonic acid in microglia.

Inflammation of LPS-stimulated microglia has various molecular mechanisms (Chen et al. 2018). Typically, the inflammation pathway concludes p38 MAPK/NF-κB pathways. These pathways play a role in the inflammatory mechanisms for microglia activation. MAPK includes serine/threonine kinases and modulates the expression of pro-inflammatory mediators and cytokines in microglia (Kaminska 2005). Lipopolysaccharide (LPS) activates inflammation signaling by toll-like receptor 4 (TLR4). Activation of TLR4 stimulates intracellular molecules of phosphorylated IκB-α and activates the MAPK family. After the phosphorylation of IκB-α, NF-κB is activated and has a role as a transcription factor (Kawai and Akira 2007; Lu et al. 2008). Activated MAPKs and NF-κB can increase the inflammatory response to microglia activation. Therefore, the regulation of microglia activation is considered to prevent inflammation, and may be a potential therapy for neuroinflammatory diseases (von Bernhardi et al. 2015). Here, we confirmed the effect of malonic acid on *in vitro* anti-inflammatory activity in LPS-activated microglia cells. Moreover, we investigated whether malonic acid regulates the expression of inflammatory mediators and cytokines in activated microglia by reducing the p38 MAPK/NF-κB pathway.

## Materials and methods

### Cell culture and chemical

The murine BV2 microglial cell line was obtained from the Korea Cell Line Bank. The cells were cultured in Dulbecco's Modified Eagle’s Medium (Welgene, Korea) containing 10% fetal bovine serum (Corning Costar, USA) and 1% antibiotic-antimycotic (Gibco, USA) in a 37°C incubator in an atmosphere of 5% CO_2_. Malonic acid was purchased from Tokyo Chemical Industry Co., Ltd. Malonic acid was dissolved in dimethyl sulfoxide (DMSO).

### Cell viability assay

Cell viability was determined by an WST-8 assay kit (Biomax.Ltd, Korea). The BV2 microglia cells were seeded into 96-well plates (1 × 10^4^ cells/well) and incubated overnight. The cells were treated with various concentrations of malonic acid for 1 h and then stimulated with LPS (200 ng/mL) for 24 h. Then, the cells were incubated with 10 μl of WST-8 solution and maintained for 3 h in a 37°C incubator in an atmosphere of 5% CO_2_. The cell viability percentage was measured using an ultraviolet spectrophotometer at 450 nm.

### Nitric oxide assay

The BV2 microglia cells were seeded into 96-well plates (1 × 10^4^ cells/well) and pre-treated with malonic acid for 1 h and stimulated with LPS (200 ng/mL) for 24 h. The collection of culture medium was assayed for NO production using a Griess reagent kit (Thermo Fisher Scientific, USA). Then, the supernatants were read at 540 nm using an ultraviolet spectrophotometer. Nitrite concentrations were calculated according to a nitrite standard curve.

### Reactive oxygen species (ROS) assay

The ROS assay in BV2 microglia cells was measured by a ROS detection assay kit (BioVision) according to the manufacturer’s protocol. The BV2 microglia cells were seeded into 96-well black plates (1 × 10^4^ cells/well) for 24 h, and malonic acid was added to the plates for 1 h followed by LPS (200 ng/mL). The production of the ROS was measured at excitation and emission wave lengths of 495 and 529 nm using a fluorescence microplate reader.

### Reverse transcriptase-polymerase chain reaction (RT–PCR) and quantitative real-time PCR analysis

The BV2 microglia cells were incubated for 24 h with LPS alone or after 1 h of pre-treatment with malonic acid. Total RNA was isolated using RNAiso Plus reagent (TaKaRa Bio, Japan), and cDNA was synthesized using cDNA MasterMix (ToYoBo, Japan) according to the manufacturer’s instructions. Quantitative real-time PCR was conducted using the AMPIGENE® qPCR Green Mix Hi-ROX (Enzo, USA) according to the manufacturer’s instructions. The mRNA expression of iNOS, IL-1β, IL-6, and TNF-α was normalized to glyceraldehyde-3-phosphate dehydrogenase (GAPDH). The primer sequences used in this study were as follows: iNOS, 5ʹ-CGGGTTGAAGTGGTATGCAC-3ʹ (forward) and 5ʹ-GCTGTGTGGTGGTCC ATGAT-3ʹ (reverse); IL-1β, 5ʹ-GTGTCTTTCCCGTGGACCTT-3ʹ (forward) and 5ʹ-TCGTT GCTTGGTTCTCCTTG-3ʹ (reverse); IL-6, 5ʹ-CCTTCCTACCCCAATTTCCA-3ʹ (forward) and 5ʹ-CGCACTAGGTTTGCCGAGTA-3ʹ (reverse); TNF-α, 5ʹ-GGCCTCTCTACCT TGTTGCC-3ʹ (forward) and 5ʹ-TAGGCGATTACAGTCACGGC-3ʹ (reverse); GAPDH, 5ʹ-TGCACCACCAACTGCTTAG-3ʹ (forward) and 5ʹ-GGATGCAGGGATGATGTTC-3ʹ (reverse).

### Western blotting

BV2 microglia cells were lysed to extraction protein in RIPA buffer. After incubation at 4°C for 35 min, each sample was centrifugated at 13,200 rpm at 4°C for 25 min. The protein concentration was measured by the bovine serum albumin (BSA) Protein Assay (Thermo Fischer Scientific, USA). A defined quantity of total protein was subjected to 10–12% SDS-PAGE gels and the resolved protein bands were transferred from gel to PVDF membranes. Each membrane was blocked using 5% skim milk in 0.05% Tween-20 with 1 x PBS (PBST). Each membrane was incubated overnight at 4°C with primary antibody diluted to 1:1000 in 5% BSA in PBST buffer. Each membrane was exposed to a secondary antibody diluted to 1:5000 for 1 h 30 min. Bands were visualized by chemiluminescence (Bio-Rad, Korea). GAPDH was used as a loading control in western blotting.

### Antibodies

The monoclonal antibodies used were against anti-NOS2 (iNOS), anti-NF-κB, anti-Lamin A/C, anti-p-IκB-α, anti-IκB-α, anti-p-JNK, anti-JNK, anti-p-p38, anti-p-38, anti-p-ERK, and anti-ERK (Santa Cruz Biotechnology). The polyclonal antibodies were against GAPDH (Bioworld Technology, USA).

### ELISA

The protein levels of IL-6 in culture medium were determined using an ELISA kit (Abbkine, China). BV2 microglia cells were pre-treated with malonic acid for 1 h and stimulated with LPS (1 μg/mL) for 24 h. The culture medium was collected and the concentration of IL-6 was measured according to the manufacturer’s protocol. Absorbance at 450 nm was determined using an ultraviolet spectrophotometer.

### Nuclear fraction

Nuclear extracted samples were prepared from BV2 microglia cells based on previously described methods (Kang et al. 2020). The protein concentration was measured by the BSA Protein Assay (Thermo Fischer Scientific, USA).

### Statistical analysis

Analysis of data was performed using GraphPad Prism5 software (GraphPad, USA). Statistical analyses were undertaken using Student’s t-test. A *P*-value less than 0.05 was considered statistically significant.

## Results

### Effect of malonic acid on the viability of microglia cells

**Figure 1.**
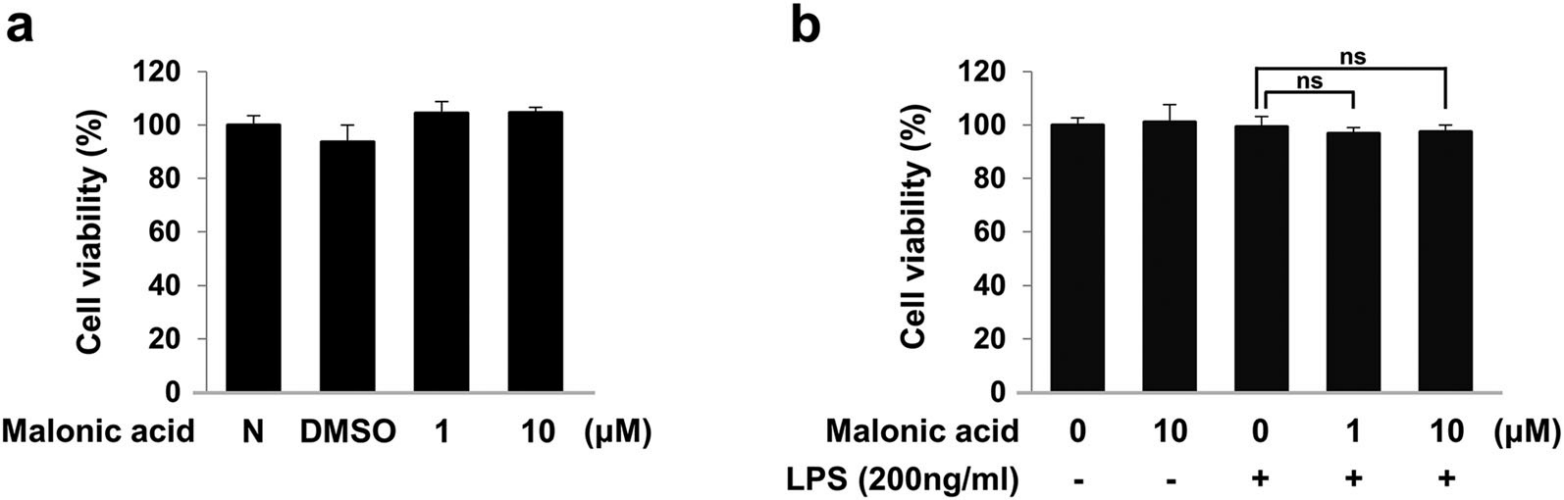
Cell viability by malonic acid in BV2 microglia cells. (a) Effects of malonic acid on cell viability in BV2 microglia cells were assessed by WST-8 assay. BV2 microglia cells were treated with 1 and 10 μM malonic acid for 24 h. (b) Effects of malonic acid and LPS on cell viability. BV2 microglia cells were pre-treated with malonic acid for 1 h and treated with LPS for 24 h. The data were analyzed in triplicate and presented as the means ± SEM. ns: not significant.

In the present study, we assessed whether malonic acid reduced inflammation induced by LPS in BV2 microglia cells. BV2 microglia cells were pre-treated with various concentrations of malonic acid (0.1–100 μM) for 1 h before LPS (200 ng/mL). 1 and 10 μM malonic acid were able to reduce inflammation activated by LPS in BV2 microglia cells (Suppl. Fig. 1). Therefore, concentrations of 1 and 10 μM malonic acid were used for our study. To determine whether malonic acid influences the cell viability of BV2 microglia cells, a WST-8 assay was performed with malonic acid for 24 h. Treatments of malonic acid had no cytotoxic effects (Figure 1(a)). Furthermore, pre-treatment with malonic acid for 1 h before LPS treatment did not affect cell viability (Figure 1(b)).

### Malonic acid reduces the production of ROS and NO release in LPS-activated microglia cells

**Figure 2.**
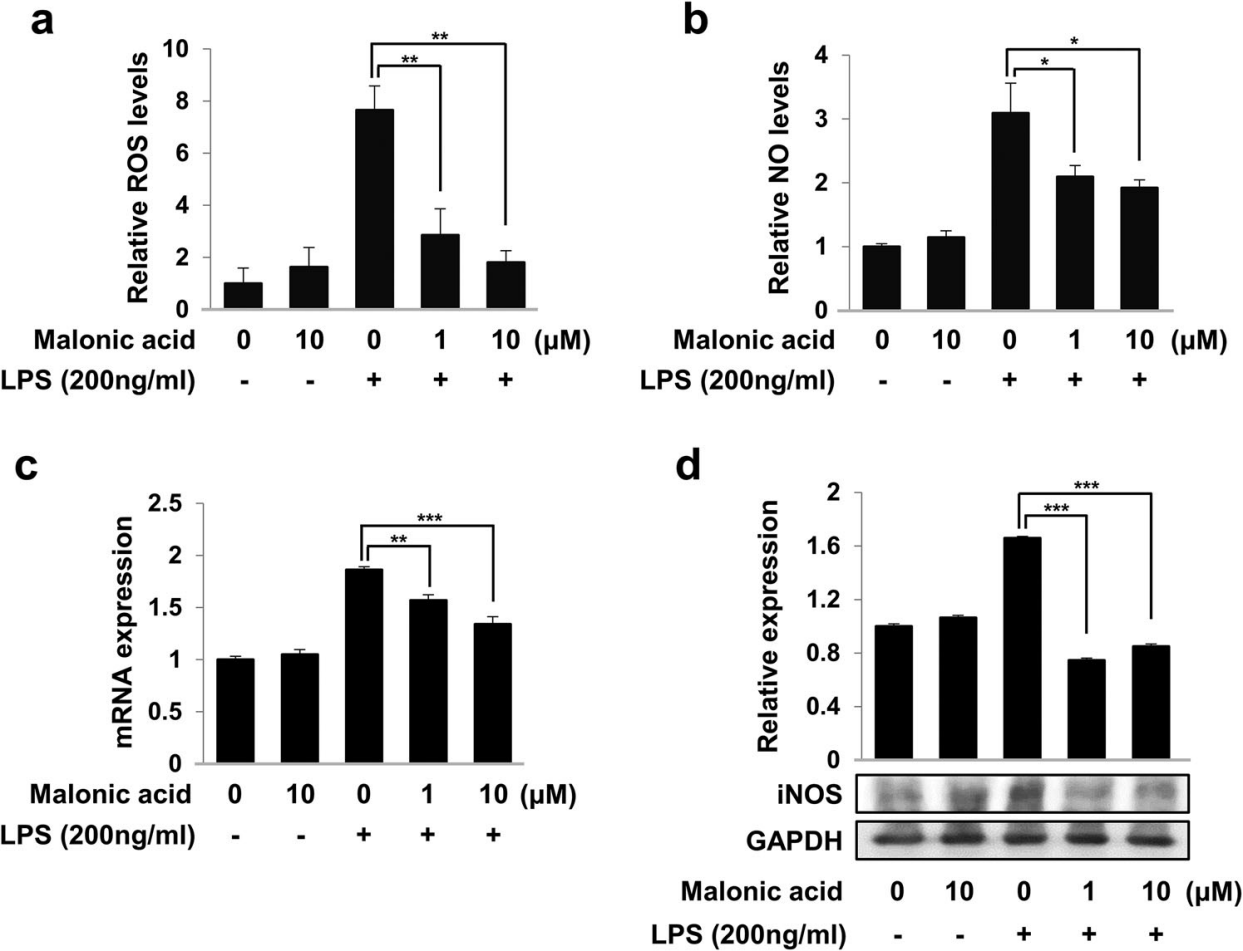
Effects of malonic acid on NO and ROS production in LPS-treated BV2 microglia cells. BV2 microglia cells were pre-treated with malonic acid for 1 h, then incubated with LPS for 24 h. (a) The amount of NO production in culture medium was detected by Griess reagent. ROS production was determined by DCFH-DA measurements. (b-c) BV2 microglia cells were treated in the same manner described above. The mRNA expression of iNOS was measured by real-time PCR (b) and the protein expression of iNOS was detected by western blot (c). The data were analyzed in triplicate and presented as the means ± SEM. *P < 0.05, **P < 0.01, ***P < 0.001.

We investigated whether the effects of malonic acid regulate the production of ROS and NO in LPS-activated BV2 microglia cells. BV2 microglia cells were pre-treated with malonic acid for 1 h and treated with LPS for 24 h. The production of ROS was analyzed using a DCFH-DA measurement kit. The levels of ROS increased upon LPS treatment of BV-2 microglia cells, whereas pretreatment of malonic acid for 1 h decreased ROS of LPS-stimulated BV2 microglia cells (Figure 2(a)). Additionally, the production of NO in the culture medium was measured by Griess reagent. Malonic acid markedly inhibited LPS-activated NO production in BV2 microglia cells (Figure 2(b)). In addition, real-time PCR and western blot analysis were performed to determine the expression of iNOS. Malonic acid effectively reduced the mRNA levels of iNOS (Figure 2(c)). Additionally, the protein expression of iNOS decreased as a result of malonic acid in LPS-treated BV2 microglia cells (Figure 2(d)). The results suggested that malonic acid effectively inhibited the production of NO and ROS activated by LPS in BV2 microglia cells.

### Malonic acid reduces mRNA levels of pro-inflammatory cytokines in LPS-activated microglia cells

**Figure 3.**
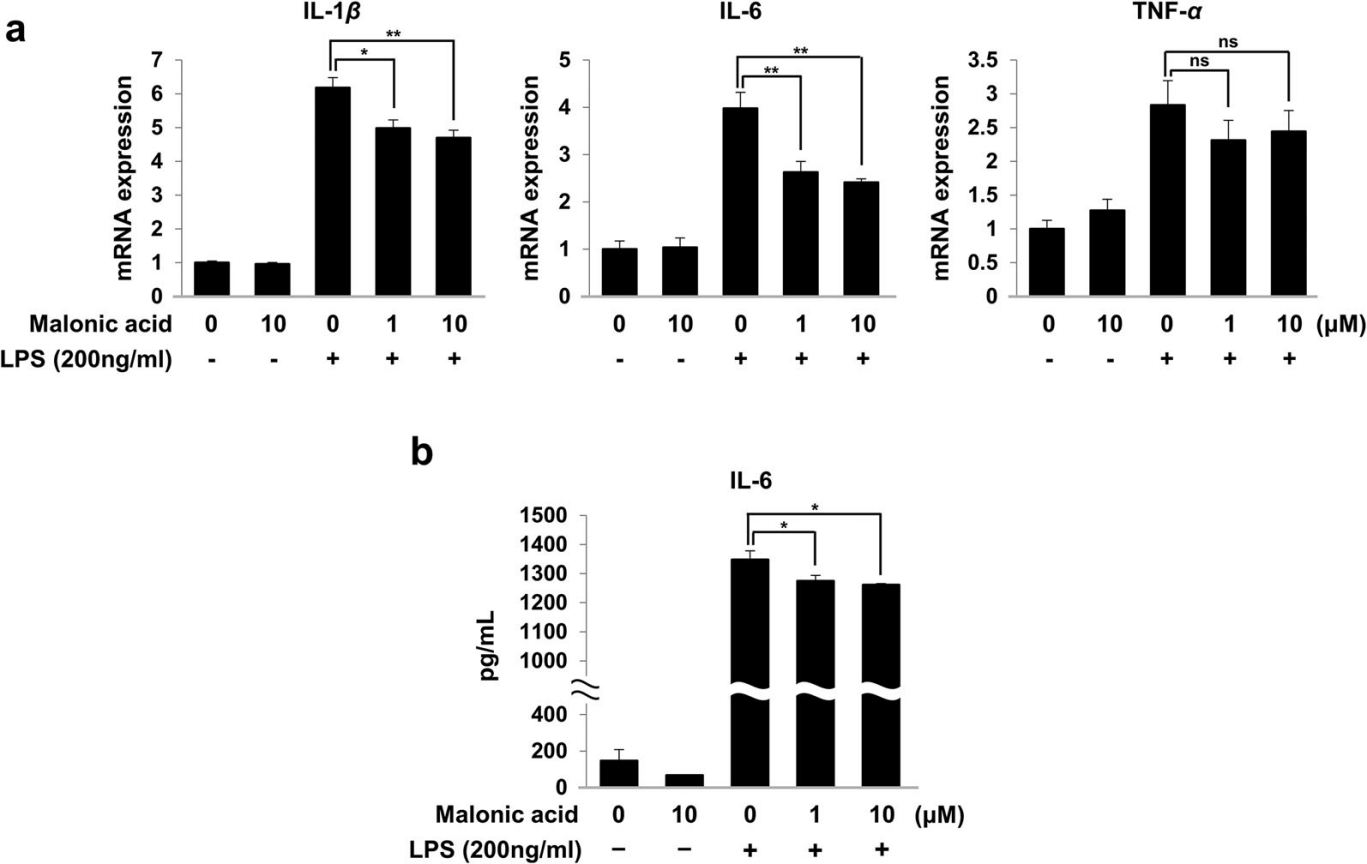
Inhibition of inflammatory mediator by malonic acid in LPS-stimulated BV2 microglia cells. BV2 microglia cells were pre-treated with malonic acid for 1 h and stimulated with LPS for 24 h. (a) The mRNA expression of IL-1β, IL-6, and TNF-α was measured by real-time PCR. (b) IL-6 released into the culture medium was quantified by ELISA. The data were analyzed in triplicate and presented as the means ± SEM. *P < 0.05, **P < 0.01, ns: not significant.

Various pro-inflammatory cytokines, including IL-1β, IL-6, and TNF-α, are major factors of activated microglia (Smith et al. 2012). We investigated whether malonic acid reduced pro-inflammatory cytokine expression in LPS-treated BV2 microglia cells. Pre-treatment with malonic acid significantly regulated the mRNA expression of IL-1β and IL-6 in LPS-treated BV2 microglia cells but TNF-α expression was not significantly regulated by malonic acid in LPS-treated BV2 microglia cells (Figure 3(a)). From the above results, mRNA expression of pro-inflammatory cytokines was reduced by malonic acid on LPS-activated microglia. The mRNA expression of IL-6 was distinctly lower compared to other cytokines. It was reported that IL-6 overproduction in the brain leads to neurodegeneration (Rothaug et al. 2016). To determine whether malonic acid inhibited not only mRNA levels but also the production of IL-6 release on LPS-stimulated BV2 microglia cells, the production of IL-6 was regulated upon pre-treatment of malonic acid before treatment of LPS in BV2 microglia cell culture medium (Figure 3(b)). The results showed the most dominant control of IL-6 in malonic acid-treated BV2 microglia cells induced by LPS.

### Effects of malonic acid on LPS-activated NF-κB-related protein expression

**Figure 4.**
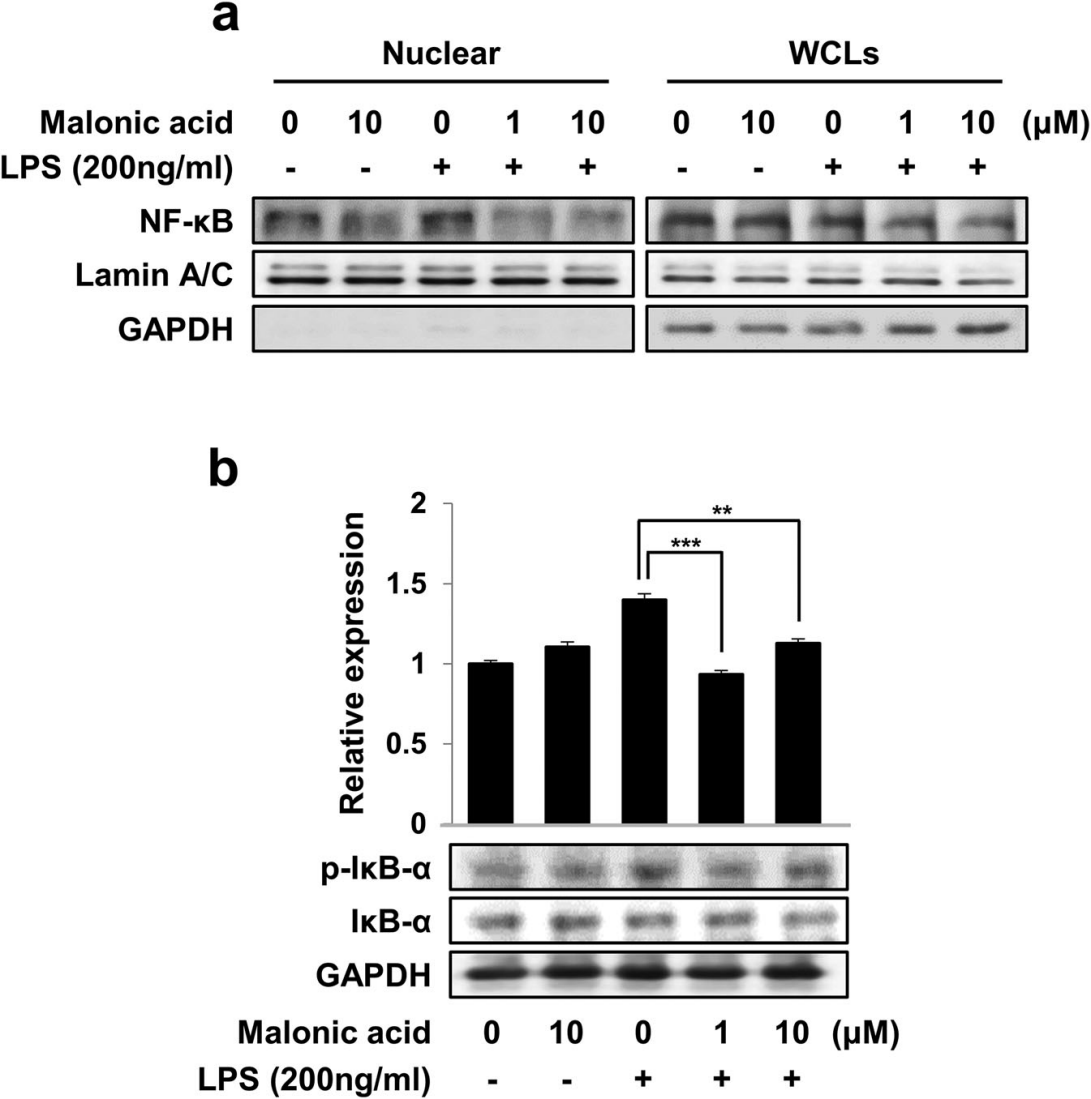
Effects of malonic acid on LPS-mediated activation of NF-κB. BV2 microglia cells were pre-treated with malonic acid for 1 h, followed by LPS treatment for 24 h. (a) Nuclear protein expression of NF-κB was detected in a nuclear fraction. The Lamin A/C was used as a control in the nuclear fraction. (b) The protein expression of p-IκB-α and IκB-α was measured by western blot. The data were analyzed in triplicate and presented as the means ± SEM. **P < 0.01, ***P < 0.001.

When NF-κB is activated by LPS, it can relocate from the cytoplasm to the nucleus and play a role as a transcription factor (Lawrence 2009). We investigated whether the effects of malonic acid regulated the translocation of NF-κB using nuclear fraction analysis. The protein expression of NF-κB in the nucleus increased after treatment with LPS. However, the treatment of malonic acid inhibited NF-κB translocation in LPS-stimulated BV2 microglia cells (Figure 4(a)). NF-κB and IκB-α coexist in the cytoplasm under normal conditions. If BV2 microglia cells are stimulated by LPS, IκB-α can undergo phosphorylation and degradation. Moreover, NF-κB is relocated from the cytoplasm to the nucleus (Liu et al. 2017). Malonic acid significantly decreased the protein expression of phosphorylated IκB-α by LPS treatment (Figure 4(b)). These results suggested that malonic acid can significantly inhibit the phosphorylation levels of IκB-α and transport NF-κB from the cytoplasm to the nucleus.

### Malonic acid reduced the activation of the p38 MAPK/NF-κB pathway in LPS-activated microglia cells

**Figure 5.**
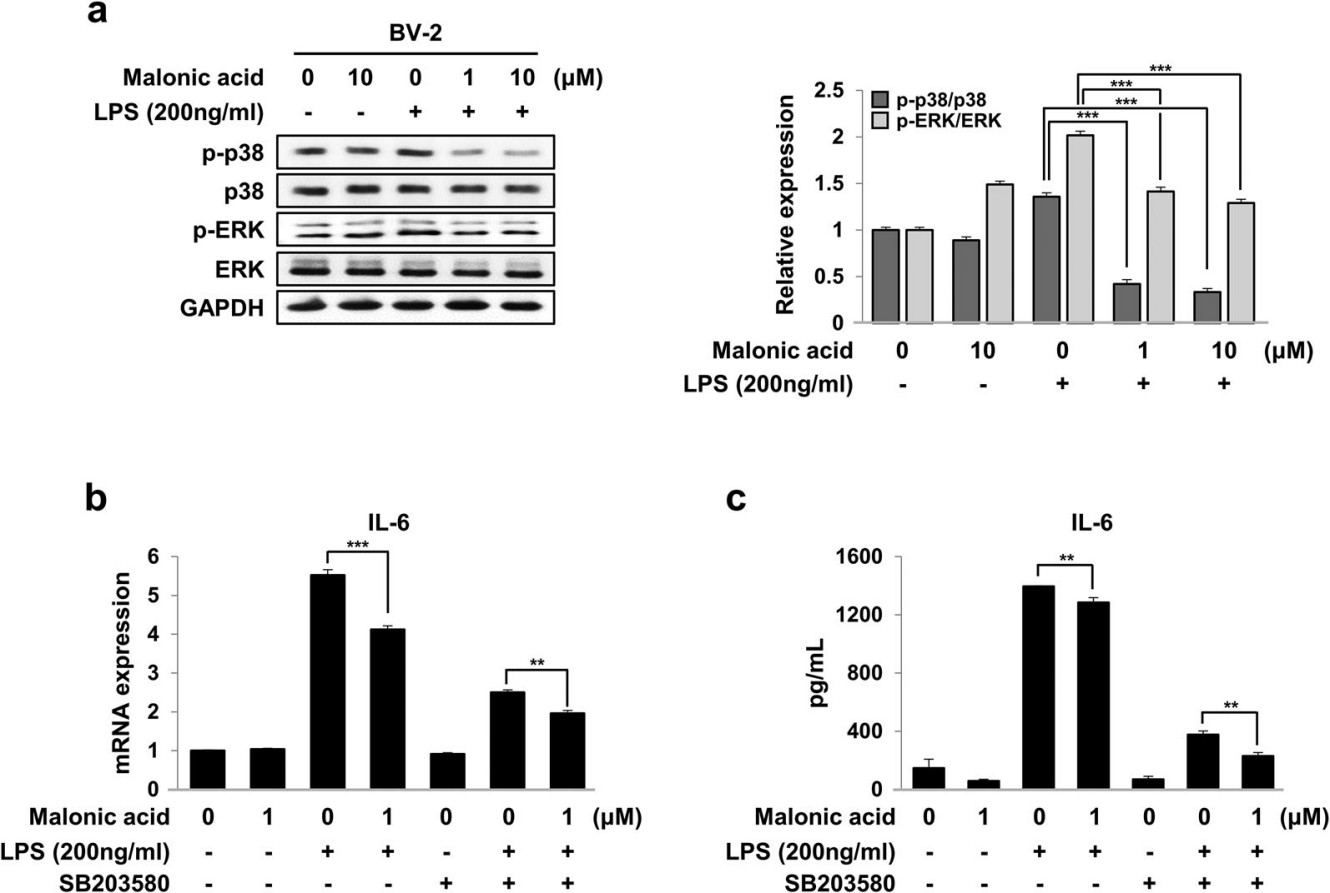
Inhibition of malonic acid on the p38 MAPK pathway in LPS-treated BV2 microglia cells. BV2 microglia cells were pre-treated with malonic acid for 1 h, followed by incubation with LPS for 24 h. (a) The protein expression of p-p38, p38, p-ERK, and ERK was detected by western blot. p38 and ERK were used as the control of phosphorylated p38 MAPK and ERK. (b-c) BV2 microglia cells were pre-treated with 1 μM malonic acid and 10 μM SB203580 for 1 h before treatment with LPS for 24 h. The mRNA levels of IL-6 were measured via real-time PCR (b) and IL-6 released into the culture medium was quantified by ELISA (c). The data were analyzed in triplicate and presented as the means ± SEM. **P < 0.01, ***P < 0.001, ns: not significant.

MAPK signaling is a major regulator of pro-inflammatory cytokines in microglia (Kaminska 2005). We determined whether malonic acid has an effect on reducing the MAPK signaling pathway in LPS-stimulated BV2 microglia cells. Western blotting suggested that malonic acid reduced the protein levels of phosphorylated ERK and p38 MAPK (Figure 5(a)). The effects of malonic acid on LPS-treated BV2 microglia cells could regulate inflammation through the p38 MAPK/ERK pathways. The mRNA expression and cytokine production of IL-6 increased by combining p38 MAPK and NF-κB (Craig et al. 2000). To further investigate whether inflammatory cytokines of IL-6 are dependent on the p38 MAPK pathway, SB203580 (a p38 MAPK inhibitor) was pre-treated for 1 h in LPS-stimulated BV2 microglia cells. SB203580 decreased LPS-stimulated IL-6 activation, thus, demonstrating that IL-6 regulation is p38 signaling pathway in BV2 microglia cells. Finally, we observed that cotreatment with malonic acid and SB203580 reduced IL-6 activation. Pre-cotreatment of malonic acid and SB203580 for 1 h before treatment of LPS was more effective at reducing the mRNA expression and cytokine production of IL-6 than treatment with SB203580 alone (Figure 5(b,c)). Although MA showed weaker effect than SB203580, but MA significantly reduced the IL-6 levels via p38 pathway. These results conclude that malonic acid reduced the LPS-induced inflammation in BV2 microglia cells via the p38 pathway.

## Discussion

Activated microglia excessively secrete various inflammatory mediators and act as cytotoxic substances, accelerating the inflammatory response and contributing to the development of chronic diseases (Kim and Joh 2006; Smith et al. 2012). Therefore, it is recognized that the generation of inflammatory mediators generated from activated BV2 microglia cells and the expression control of related genes are useful for the treatment and prevention of various diseases. Recently, natural compounds have been investigated for their anti-inflammatory abilities and potential use as preventive agents of neurodegenerative diseases (Srivastava and Yadav 2016). However, of the natural compounds, the mechanism of malonic acid on microglia is not yet fully understood. Therefore, in this study, we suggest that malonic acid inhibits the production of the various inflammatory mediators and cytokines in LPS-activated microglia. Additionally, we showed that malonic acid regulated the LPS-activated phosphorylation of p38 MAPK and the activation of NF-κB.

LPS is commonly used for the activation of inflammation through TLR4 on microglia (Lu et al. 2008). The anti-inflammatory effects of malonic acid were investigated in LPS-activated microglia. Malonic acid regulates inflammation by measuring mRNA levels of IL-6 and cytotoxic effects. Malonic acid has anti-inflammatory effects in a dose-dependent manner and has no cytotoxic effects on microglia. A low concentration of malonic acid was used in this study, and it shows sufficiently anti-inflammatory effects on the treatment of malonic acid 1 and 10 μM in LPS-activated microglia. Additionally, excessive NO secretion *in vivo* causes cytotoxicity, promotes inflammatory reactions, and mediates inflammatory reactions in the CNS, thereby participating in the development of degenerative brain diseases (Paakkari and Lindsberg 1995; Tripathi et al. 2007). Therefore, we confirmed the effect of malonic acid treatment on NO and ROS production. Through the results, malonic acid significantly reduced ROS and NO generated from BV2 cells activated by LPS treatment, which inhibits the transcription step of the NO synthase iNOS gene. We confirmed that malonic acid on LPS-activated microglia efficiently inhibit various inflammatory mediators and pro-inflammatory cytokines. Activated microglia secrete cytokines, such as IL-1β, IL-6, IL-10, TGF-β, and TNF-α, which are expressed at very low levels under normal conditions, but are highly expressed under excessively damaged conditions to promote inflammation (Smith et al. 2012; Kany et al. 2019). In particular, IL-1β, IL-6, and TNF-α are representative pro-inflammatory cytokines associated with various degenerative diseases (Chen et al. 2016). According to the results, malonic acid regulates the gene expression of IL-1β and IL-6 in the LPS-activated BV2 cells. Additionally, the secretion of IL-6 is regulated by malonic acid treatment. However, TNF-α was not significantly reduced, but it was confirmed that the pattern was decreasing. These findings are thought to indicate the possibility that malonic acid can specifically regulate cytokine. However, this requires further study.

In the inflammation pathway of microglia, NF-κB for transcription factor and MAPK pathways is a component of JNK, ERK, and p38 MAPK pathway-induced mRNA and protein levels of various inflammation factors (Hanisch 2002; Kaminska 2005; Lawrence 2009; Liu et al. 2017). Our results showed that malonic acid decreases protein levels of phosphorylated NF-κB and p38/ERK. However, malonic acid did not regulate phosphorylation levels of JNK signaling. Previous studies have investigated whether natural food of royal jelly regulate LPS-activated inflammation in microglia via the NF-κB and p38/JNK pathway. Although royal jelly does not regulate protein levels of phosphorylated ERK, which efficiently showed anti-inflammatory effects on LPS-activated microglia (You et al. 2018). Similar to the above, malonic acid also regulated inflammation on microglia through a major target to the p38 pathway compared to the JNK and ERK pathway. Interestingly, a recent study suggested that bis-malonic acid fullerene derivative in BMMC inhibited the activation of NF-κB but not p38 MAPK and JNK pathways (Funakoshi-Tago 2016). Malonic acid on LPS-activated microglia can regulate the phosphorylated protein expression of p38 and ERK. It possibly shows that different cell lines make different signaling pathways, and we assumed that malonic acid may be respectively inhibited not only by NF-κB activation but also p38 phosphorylation in microglia cells. Therefore, we reveal that malonic acid can suppresses the phosphorylation of p38 and activation of NF-κB. In microglia, the activation of IL-6-promoting LPS was regulated by collaborated expression of NF-κB and p38 pathway (Craig et al. 2000). Moreover, promoter sequences of IL-6 contain that which are recognized by transcription factors of NF-κB (Libermann and Baltimore 1990). This process is relative to the activation to initiate neuroinflammation (Newton and Dixit 2012). These results show that malonic acid reduces inflammation through the regulation of IL-6 mRNA and protein expression, and that IL-6 mediated inflammation is possibility regulated through NF-κB and p38 MAPK pathways by malonic acid.

In summary, we investigated the anti-inflammatory function and related mechanisms of malonic acid. According to the results, we demonstrated the effect of malonic acid on the production of NO, which are important inflammatory mediators induced by LPS in BV2 microglia cells. It was found that this is due to the blocking of expression of iNOS. In addition, malonic acid significantly inhibited the production of IL-6 and IL-1β, which are inflammatory cytokines whose levels are increased by LPS, which was found to be due to blocking the expression of these genes at the transcription level. In particular, the downregulated activation of NF-κB and p38 MAPK pathways reduces the downstream of IL-6 activation. This may indicate the possibility of therapeutic effects against neuroinflammation.

## Supplementary Material

Supplemental Material
