# Supplementary material for: Malonic acid suppresses lipopolysaccharide-induced BV2 microglia cell activation by inhibiting the p38 MAPK/NF-κB pathway
Source: Anim Cells Syst (Seoul). 2021 Mar 18;25(2):110–8. doi: 10.1080/19768354.2021.1901781 (PMC8118420; doi:10.1080/19768354.2021.1901781)
Supplement: Supplemental Material [file TACS_A_1901781_SM7773.docx]

**Supplementary figure legends**

**
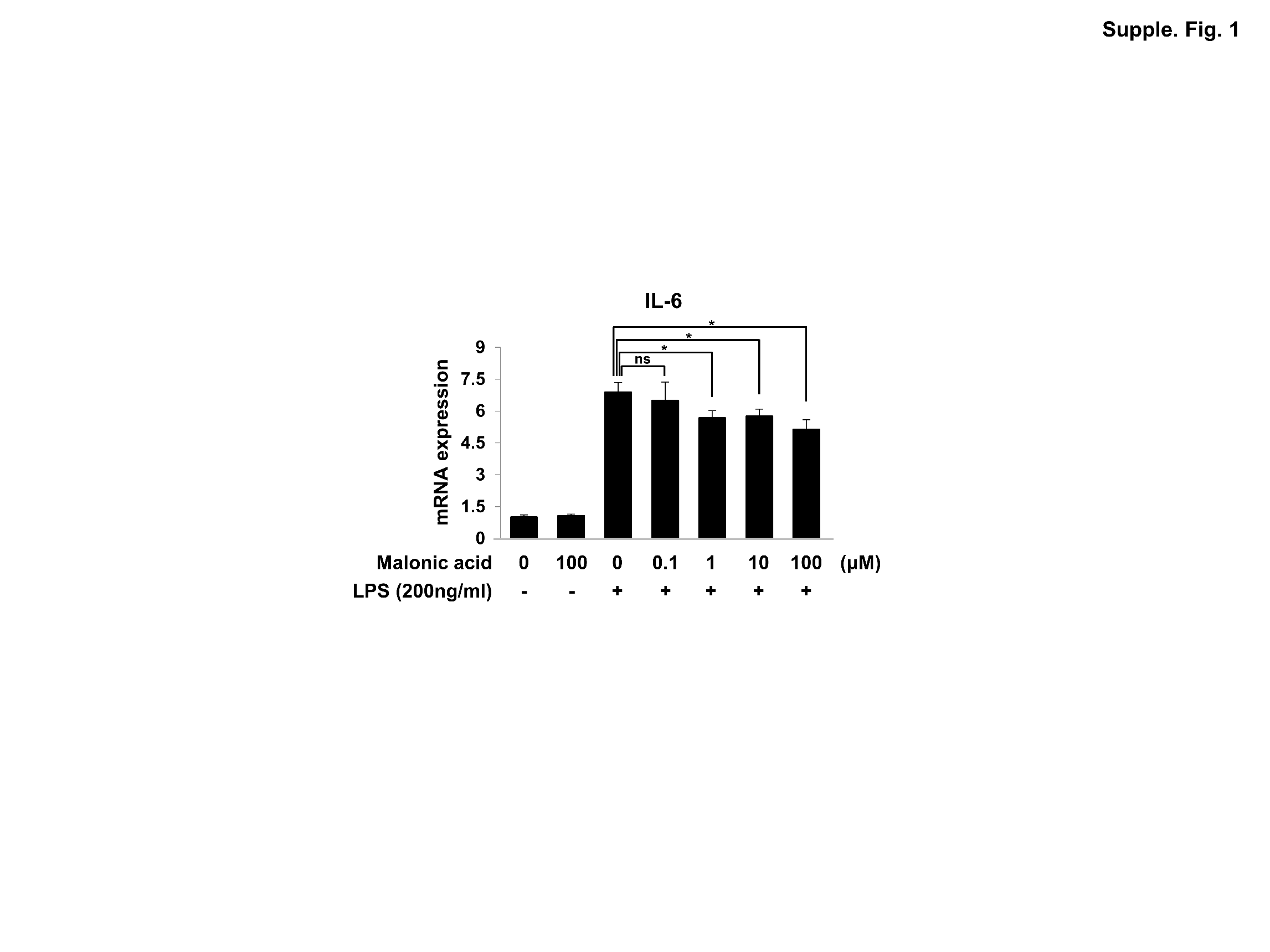
**

**Supplementary Fig. 1** Effect of various concentrations of malonic acid on IL-6 levels in LPS-stimulated BV2 microglia cells. Effects of malonic acid on the anti-inflammatory ability in LPS-activated BV2 microglia cells. BV2 microglia cells were treated with 0.1, 1, 10, and 100 μM malonic acid for 1 h before treatment with LPS for 24 h. mRNA levels of IL-6 were measured by real-time PCR. The data were analyzed in triplicate and presented as the means ± SEM. ^*^*P* < 0.05.
